# Chronic Heel Pain Found to Be Diffuse Large B-cell Lymphoma of the Calcaneus

**DOI:** 10.7759/cureus.25282

**Published:** 2022-05-24

**Authors:** Kevin J Horner, Caitlyn J Smith, Katsiaryna Laziuk, Benjamin Summerhays

**Affiliations:** 1 Orthopaedics, University of Missouri School of Medicine, Columbia, USA; 2 Pathology and Anatomical Sciences, University of Missouri School of Medicine, Columbia, USA

**Keywords:** primary bone lymphoma, dlbcl, heel pain, calcaneus, lymphoma

## Abstract

Primary non-Hodgkin lymphoma of the bone is quite rare, accounting for less than 2% of all lymphomas in adults. We present a unique case of chronic left heel pain in a 34-year-old pregnant woman with a remote history of lower extremity trauma. Unresponsive to conservative treatment, advanced imaging showed an infiltrative soft tissue mass involving the calcaneus and surrounding soft tissues. Biopsy of the area confirmed the diagnosis of Stage I-AE diffuse large B-cell lymphoma. The patient underwent 20 localized radiation treatments to the left heel. Nine years after the diagnosis, the patient remains in remission without signs of disease recurrence. This case report demonstrates that atypical and less common causes of chronic heel pain should be considered in certain clinical scenarios, especially in those unresponsive to conservative treatment.

## Introduction

Heel pain is most commonly the result of a mechanical etiology. Clinical practice guidelines further subcategorize mechanical heel pain into plantar and posterior heel pain, with plantar heel pain being the most prevalent complaint [1]. Despite the commonality of certain diagnoses, neurologic, arthritic, traumatic, and other etiologies of heel pain should be considered. Although quite rare, benign and malignant neoplasms of the calcaneus have been described [2-12]. Furthermore, primary bone lymphoma of the calcaneus is seldom reported [7-12]. In this article, we report an uncommon case of diffuse large B-cell lymphoma (DLBCL) involving the calcaneus.

## Case presentation

A 34-year-old, 14-week pregnant African American female was referred to the senior author for chronic left ankle, heel, and foot pain. Her medical history was significant for hypertension, diabetes mellitus type 2, iron deficiency anemia, obstructive sleep apnea, polycystic ovarian syndrome, and obesity (body mass index: 52 kg/m^2^). She denied using tobacco products and alcohol, had no known drug allergies, and was taking ondansetron, ferrous sulfate, labetalol, ranitidine, and glyburide at the time of presentation. There was no reported family history of cancer.

She reported an injury to the left lower extremity when she fell through a floor six years earlier and had also been in a car accident 16 months prior. Both incidents incited pain to the left foot. She had been unresponsive to conservative treatments, including physical therapy, pain medication, a controlled ankle movement (CAM) boot, trials of non-weight-bearing status, a total contact cast, and an ultrasound bone healing system. After failing conservative treatment, she was referred to the senior author for further evaluation and treatment. Continued pain and failure to respond to treatment prompted additional imaging and clinical follow-up.

Physical examination of the left foot revealed obvious swelling and deformity to the hindfoot and calcaneus with detectable warmth of the foot. She was tender to palpation surrounding the left hindfoot and calcaneus. The passive range of motion of the ankle was full, and the foot was neurovascularly intact.

**Figure 1:**
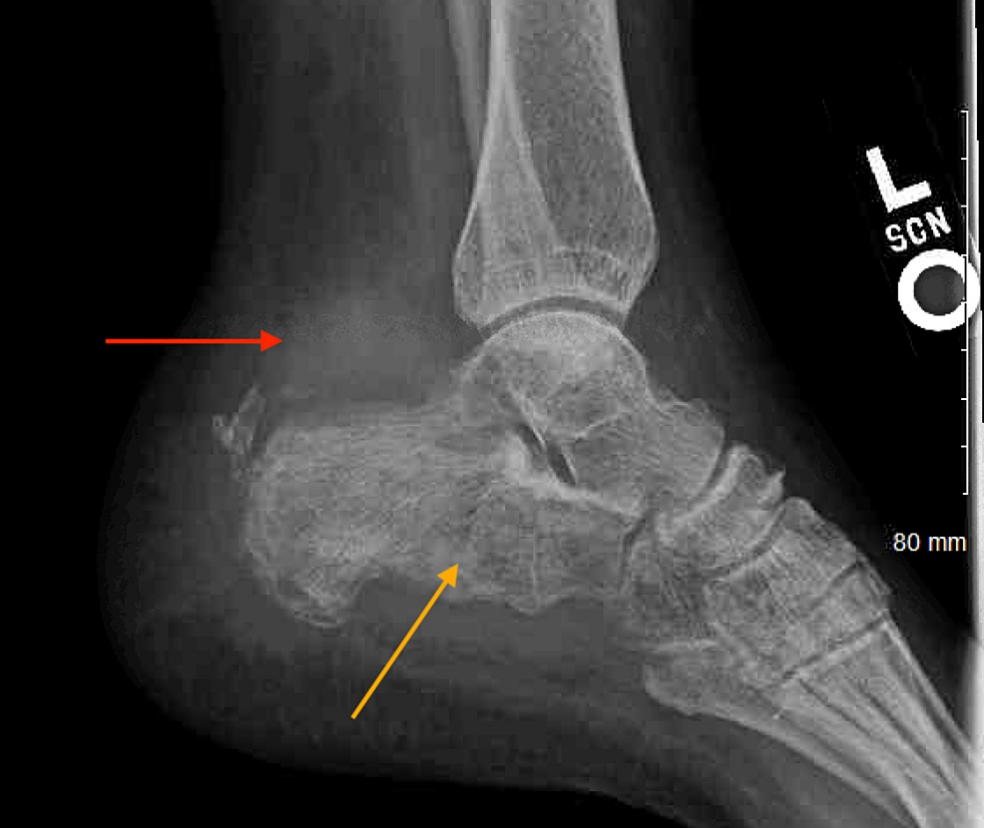
Left ankle radiograph. Left ankle radiograph showing an infiltrative soft tissue mass involving the calcaneus and adjacent posterior soft tissues. Permeative osteolysis is present (red arrow), and the calcaneus has a mottled appearance (orange arrow).

**Figure 2:**
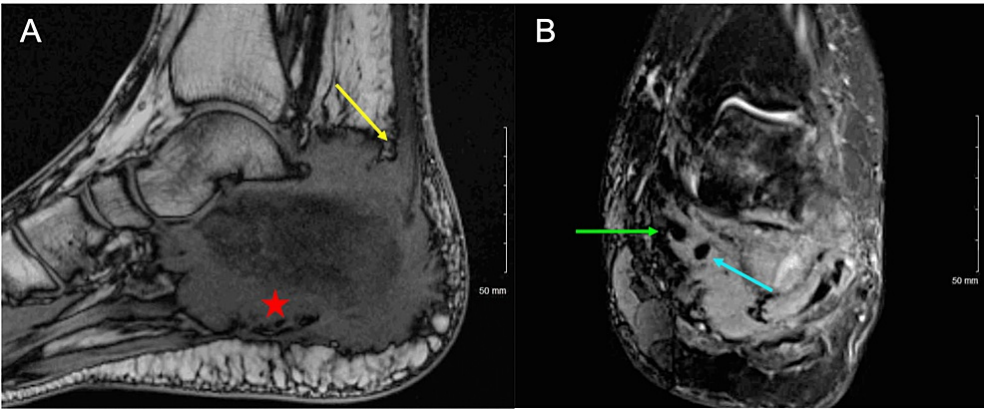
Multisequence, multiplanar left ankle magnetic resonance imaging without contrast. (A) Sagittal T1 image showing a circumferential soft tissue mass of the calcaneus with irregular cortical disruption. There is also involvement of the Achilles tendon insertion (yellow arrow) and posterior plantar fascia (red star). (B) Coronal short tau inversion recovery image showing the mass to completely encase the flexor hallucis longus tendon (blue arrow). The mass also abuts the deep margins of the flexor digitorum longus tendon (green arrow).

Updated radiographs revealed an infiltrative soft tissue mass involving the calcaneus and adjacent posterior soft tissues. The calcaneus had a mottled appearance, and there was no evidence of acute fracture or joint dislocation (Figure 1). Magnetic resonance imaging (MRI) of the left ankle showed a 1.5 cm thick circumferential soft tissue mass of the calcaneus with irregular cortical disruption and permeative intramedullary signal involving the entire bone. The mass was also noted to completely encase the flexor hallucis longus tendon with additional involvement of the peroneal tendons, plantar fascia, calcaneofibular ligament, and Achilles tendon insertion (Figure 2).

A decision was made to take the patient to the operating room for bone and soft tissue biopsy. Twenty-seven weeks pregnant at this time, the patient received an ankle ring block instead of general anesthesia. A 3 cm incision was made along the left lateral heel down to the bone. No purulence, abscess, or abnormal fluid was noted. Three uni-cortical calcaneal bone biopsies were obtained and sent for pathological analysis. A 2.5 cm soft tissue specimen was also sent for fresh frozen pathological analysis. Intraoperatively obtained Gram stain and bacterial and fungal cultures proved negative.

**Figure 3:**
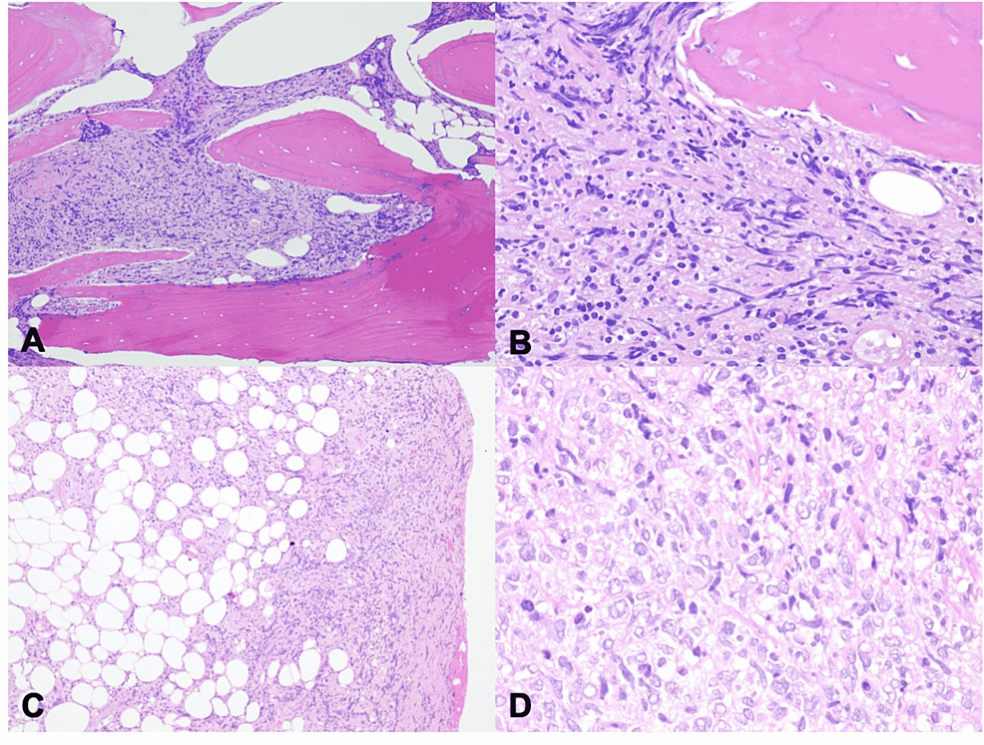
Calcaneus biopsy (A and B) and left heel soft tissue biopsy (C and D). (A) Microscopically, at low magnification (100×), hematoxylin and eosin stain showed sheets of a monotonous, large, and pleomorphic cell population infiltrating into the calcaneus. (B) At higher power (400×), tumor cells demonstrate moderately clear to slightly basophilic cytoplasm with large nuclei, occasionally vesicular, and with irregular nuclear contours. (C) At low magnification (100×), hematoxylin and eosin stain showed a monotonous population of tumor cells invading the left heel’s soft tissue. (D) Higher power (400×) demonstrates a proliferation of large, pleomorphic cells with clear basophilic cytoplasm with prominent nuclear contours, like the tumor cells seen in (B).

Macroscopically, the core biopsy of the bone of the central anterior-lateral to the medial calcaneus, 0.3 × 1.7 cm, was received fresh for the frozen section. The intraoperative diagnosis was a spindle cell tumor. Microscopic examination demonstrated that the bone and soft tissue were extensively involved by a monotonous proliferation of large and pleomorphic cells (Figures 3A, 3B). Macroscopically, the soft tissue biopsy of the left heel medial suture site measured 2.6 × 1.9 × 0.7 cm. Microscopic examination of this specimen showed the neoplasm to be invading through the marrow space, through the trabecular bone, and extending into the surrounding soft tissue of the left heel (Figures 3C, 3D).

**Figure 4:**
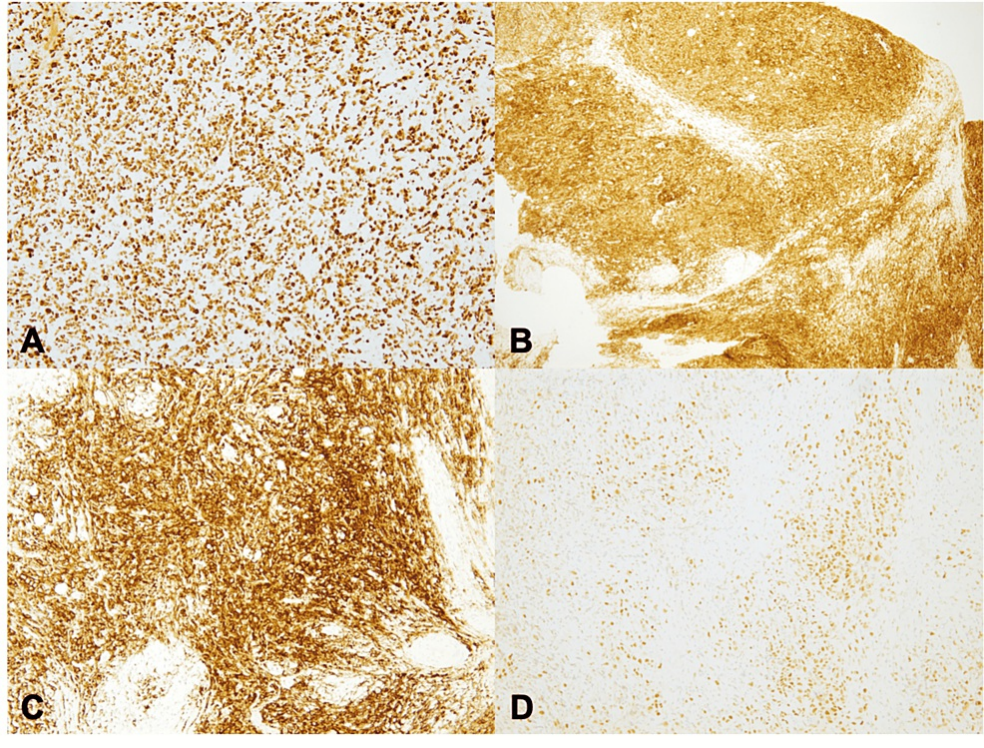
Immunohistochemistry stains. (A) Microscopically, at low magnification (100×) showed positive staining in nearly all tumor cells, approximately 100%. (B) At low magnification (100×) showed positive staining for CD45, pan-leukocyte marker, highlighting hematopoietic cells involved in the regulation and activation of the immune system. (C) The tumor cells displayed immunopositivity for CD20, a B-cell marker. (D) There was positive staining for B-cell lymphoma 6, a germinal cell marker.

At an outside facility, immunohistochemistry revealed the tumor cells staining positively for Ki-67, CD45, CD20, and B-cell lymphoma 6 (BCL-6) (Figures 4A-4D). Multiple myeloma 1 (MUM1) was noncontributory in this case. This lesion was immunophenotypically negative for CD34, terminal deoxynucleotidyl transferase (TdT), myeloperoxidase (MPO), CD30, S100, human melanoma black 45 (HMB45), neuron-specific enolase (NSE), chromogranin, synaptophysin, CD57, smooth muscle actin, cytokeratin, anaplastic lymphoma kinase C (ALK-C), epithelial membrane antigen (EMA), cyclin D1, CD117, CD68, CD4, CD8, CD3, and CD10.

Molecular studies such as fluorescent in-situ hybridization were performed to evaluate for an MYC rearrangement or possible “double hit” and showed no evidence for the IgH/BCL-2 translocation associated with t(14;18) using the IGH/BCL-2 dual-color, dual transfusion probe assay. Lack of rearrangement, in this case, ruled out other high-grade lymphomas. Flow cytometry did not demonstrate a clonal population of B cells. The diagnosis of DLBCL was based on the morphological appearance of the tumor cells and the Ki-67 index. The Ki-67 index approached nearly 100% immunopositivity in the tumor cells, which is not typical in DLBCL. The diffuse immunopositivity of BCL-6 and noncontributory MUM1 was suggestive of the germinal center B-cell-like subtype of DLBCL.

Staging MRI scans of the neck, chest, abdomen, and pelvis showed no other evidence of lymphoma, and the patient was referred to the oncology service. She was diagnosed with Stage I-AE DLBCL of the calcaneus and surrounding soft tissue. The decision was made to initiate treatment instead of waiting until after the delivery of her child. Over the course of a month in her third trimester, she underwent 20 localized radiation treatments to the left heel (40 Gy in 20 fractions). Pregnancy shielding was utilized to reduce the dose of radiation to the fetus. For the rest of her pregnancy, the patient continued minimal weight-bearing in her CAM boot secondary to pain, mobility issues, and concern of developing a pathological fracture.

After a successful full-term delivery, the oncology team completed the staging process with a formal whole-body positron emission tomography (PET) scan which showed no fluorodeoxyglucose (FDG)-avid lesions. No chemotherapy was prescribed during or after the pregnancy. Soreness around the ankle continued in the months following radiation treatment. Four years later, a repeat PET scan was negative for lymphoma. Nine years following the diagnosis, the patient is without fevers, chills, and weight loss and is displaying no signs of disease recurrence. Radiographs are stable and the oncology team will continue to observe the patient annually for physical examinations and lab work.

## Discussion

DLBCL is the most common type of non-Hodgkin lymphoma [13]. Primary non-Hodgkin lymphoma of the bone (PLB), however, is quite rare. PLB accounts for less than 2% of all lymphomas in adults and 3-7% of primary bone tumors [14,15]. Interestingly, most primary bone lymphomas are DLBCL. In their retrospective study, Horsman et al. found that 70.3% of primary bone lymphomas were DLBCL [16]. The location of primary bone lymphoma can vary. In an 82-patient study, 55% of primary cases arose in the femur, pelvis, tibia, or fibula [17]. Primary bone lymphoma of the calcaneus, however, is not very common, with very few cases reported in the literature [7-12]. The clinical presentation of primary bone lymphoma can vary in severity and duration of symptoms. Although localized pain and soft tissue swelling may be the only signs of disease, constitutional symptoms have been described [18]. Some patients may have an insidious onset of symptoms while others may have a rapidly enlarging mass [18]. It should be noted that cases of primary bone lymphoma that have a cortical breakthrough, pathological fractures, and soft-tissue involvement are more aggressive and tend to have a poorer prognosis [19].

Radiographs are likely to be the first imaging modality in the assessment of heel pain, and clinicians should be aware that osseous lymphomas most commonly take on a lytic appearance [20]. While most films would have a moth-eaten/permeative wide zone of transition, some lytic lesions may be well-defined, appearing similar to the lesions of multiple myeloma [20]. In equivocal cases of heel pain, MRI can be extremely useful to evaluate the location and extent of pathology depending on the etiology [21]. When approaching PLB, it is important to localize the tumor with imaging studies to identify if the lymphoma is a primary lesion or represents the metastatic spread of systemic lymphoma. Tissue samples from a biopsy with the subsequent histologic, molecular, and cytogenetic examination are used for the definitive diagnosis of PLB [7]. In our case, the patient underwent a core biopsy of the bone following her MRI.

The pathological diagnosis of DLBCL is made by evaluation of multiple molecular alterations, including c-MYC (codes for transcription factor), BCL-2 (oncogene), and BCL-6 (transcription repressor), that have been identified in the pathogenesis of DLBCL subtypes and have prognostic implications [22]. Simultaneous rearrangement of c-MYC, BCL-2, and BCL-6 genes are involved in double-hit lymphomas, which are high-grade B-cell lymphomas with poor prognoses [22]. DLBCL is considered a double expressor lymphoma when overexpression involves two of these genes without gene rearrangement or translocations [22].

There are currently no randomized trials assessing treatment alternatives for PLB. At present, multiagent chemotherapy with or without radiation therapy is preferred [7]. Upon review, the treatment regimens utilized in the management of calcaneal PLB have varied, using a combination of the two aforementioned treatment modalities. One patient underwent six cycles of chemotherapy without radiation therapy [8], others had cycles of chemotherapy followed by radiotherapy [7,9,12], and two more, including our patient, had localized radiation treatments only [10]. This variety highlights the need to take into account individual clinical scenarios when coming up with a treatment plan. In the case of our patient, her ongoing pregnancy at the time of diagnosis was a major factor in the decision-making process.

## Conclusions

PLB is quite rare and most cases are found to be DLBCL, which is further supported by this case report. Although cases of PLB involving the calcaneus are seldom encountered, good results have been reported despite varying treatment modalities. For many, advanced imaging will prove critical in the diagnosis of less common etiologies of heel pain. When assessing patients with heel pain, clinicians must consider the chronicity of the pain as well as the location, severity, and associated symptoms.
